# Health Services Utilization, Work Absenteeism and Costs of Pandemic Influenza A (H1N1) 2009 in Spain: A Multicenter-Longitudinal Study

**DOI:** 10.1371/journal.pone.0031696

**Published:** 2012-02-14

**Authors:** Mariana Galante, Olatz Garin, Elisa Sicuri, Francesc Cots, Anna García-Altés, Montserrat Ferrer, Àngela Dominguez, Jordi Alonso

**Affiliations:** 1 CIBER Epidemiología y Salud Pública (CIBERESP), Madrid, Spain; 2 Health Services Research Group, IMIM-Research Institute Hospital del Mar, Barcelona, Spain; 3 Universitat Pompeu Fabra, Barcelona, Spain; 4 Barcelona Centre for International Health Research (CRESIB, Hospital Clínic-Universitat de Barcelona), Barcelona, Spain; 5 Epidemiology and Evaluation Department, IMIM-Hospital del Mar, Barcelona, Spain; 6 Catalan Agency for Health Information, Assessment and Quality (CAHIAQ), Barcelona, Spain; 7 Universitat Autònoma de Barcelona, Barcelona, Spain; 8 Department of Public Health, Universitat de Barcelona, Barcelona, Spain; University of Hong Kong, Hong Kong

## Abstract

**Background:**

The aim of this study was to estimate healthcare resource utilization, work absenteeism and cost per patient with pandemic influenza (H1N1)2009, from its beginning to March 2010, in Spain. We also estimated the economic impact on healthcare services.

**Methods and Findings:**

Longitudinal, descriptive, multicenter study of in- and outpatients with confirmed diagnosis of influenza A (H1N1) in Spain. Temporal distribution of cases was comparable to that in Spain. Information of healthcare and social resources used from one week before admission (inpatient) or index-medical visit (outpatient) until recovery was gathered. Unit cost was imputed to utilization frequency for the monetary valuation of use. Mean cost per patient was calculated. A sensitivity analysis was conducted, and variables correlated with cost per patient were identified. Economic impact on the healthcare system was estimated using healthcare costs per patient and both, the reported number of confirmed and clinical cases in Spain. 172 inpatients and 224 outpatients were included. Less than 10% were over 65 years old and more than 50% had previous comorbidities. 12.8% of inpatients were admitted to the Intensive Care Unit. Mean length of hospital stay of patients not requiring critical care was 5 days (SD = 4.4). All working-inpatients and 91.7% working-outpatients went on sick leave. On average, work absenteeism was 30.5 days (SD = 20.7) for the first ones and 9 days (SD = 6.3) for the latest. Caregivers of 21.7% of inpatients and 8.5% of outpatients also had work absenteeism during 10.7 and 4.1 days on average respectively. Mean cost was €6,236/inpatient (CI95% = 1,384–14,623) and €940/outpatient (CI95% = 66–3,064). The healthcare economic burden of patients with confirmed influenza was €144,773,577 (IC95% 13,753,043–383,467,535). More than 86% of expenditures were a result of outpatients' utilization.

**Conclusion:**

Cost per H1N1-patient did not defer much from seasonal influenza estimates. Hospitalizations and work absenteeism represented the highest cost per patient.

## Introduction

Each year, seasonal influenza waves result in substantial mortality and morbidity, productivity losses, increase healthcare utilization, and costs [1]–[4]. Occasionally, the virus spread globally generating the need of rapid responses from healthcare services and public health policy makers. Such was the case of the pandemic influenza A (H1N1) 2009. Evidence based decisions in such circumstances are essential for the best management [5]. However, there is scarce information about social and healthcare resource utilization and their associated costs in both epidemic and pandemic influenza outbreaks.

Significant increases in healthcare use are expected every year due to winter-virus morbidity [4]. Characteristics of hospital resource utilization have been described for seasonal influenza in the US and UK [2], [6], [7]. In the case of influenza A (H1N1) 2009 there exist some information of admissions to critical care services [8]. Nevertheless, most flu cases will never be admitted to the hospital. For instance, 744,795 confirmed influenza A (H1N1) 2009 cases in Spain were treated in the ambulatory health services, and only 3,025 were hospitalized [9], [10].

Furthermore, the most important burden of influenza is related to productivity losses of both patients and caregivers [11]–[13]. The higher incidence among children during the pandemic might have increased the number of people who needed to be absent from work for caregiving [14]. In fact, work leave due to flu syndrome was even more frequent than that observed in previous seasonal influenza outbreaks [15].

While modelling tools can be useful to anticipate the economic impact of influenza outbreaks and their determinants, they are limited by their assumptions which concern unknown future events. Many studies published at the beginning of the pandemic flu in 2009 have overestimated the morbidity and mortality rates and hence, both pandemics economic impact and the effectiveness of massive vaccination programmes [16]–[18]. The alternative to simulations is the use of empirical studies. Badia et al estimated the seasonal economic impact of influenza in €1036.9 million in Spain more than one decade ago [1]. However, neither has recently economic impact been measured for seasonal influenza in Spain, nor have the cost determinants for pandemic influenza A (H1N1) 2009.

Increasing knowledge about resource utilization and its associated costs during the pandemic flu should facilitate decision making and planning public health policies in future outbreaks. The primary objective of this study was to estimate the healthcare resource utilization, work absenteeism and cost per patient with confirmed diagnosis of pandemic influenza A (H1N1) 2009, from the beginning of the pandemic to march 2010, in Spain. As a secondary objective, we estimated the economic impact of H1N1 cases on the healthcare services at a national level.

## Methods

This was a multicenter, observational, longitudinal study assessing healthcare resources utilization and work absenteeism of inpatients and outpatients with confirmed diagnosis of influenza A (H1N1) 2009 in Spain. We estimated the associated direct healthcare costs and indirect costs derived from work absenteeism; using an incidence approximation. Since flu consequences generally occur during a short period of time, the time horizon was four months. Hence, discount rates were not needed. Costs per patient are presented in € (2009), and they are a function of unit costs and the frequency of social and healthcare resource use. In addition, we estimated the economic burden of pandemic Influenza A (H1N1) at the overall population level from the healthcare provider's perspective. To achieve this objective we took into consideration both healthcare costs per patient and the reported number of influenza cases to the Spanish influenza Sentinel Surveillance System (outpatients) coordinated at the National Centre of Epidemiology (Institute of Health Carlos III) and to Surveillance System of severe influenza cases and deaths coordinated by the Centre for Health Alerts and Emergencies within Spanish Ministry of Health and Social Policy (inpatients) [9], [10].

### Ethics Statement

This study was approved by the Parc de Salut Mar ethic review board, and followed contemporary Spanish laws and declaration of Helsinki. Written informed consent was obtained from all patients. In the case of children and adults with mental disabilities, written consent was provided by parents/guardians.

### Estimating healthcare resource utilization and work absenteeism

Healthcare resources utilization, and work absenteeism of patients and their caregivers were directly reported by a population of inpatients and outpatients with confirmed diagnosis of influenza A (H1N1) 2009.

#### Population

Variables of interest were measured in a subsample of patients recruited for the study *“Risk factors of influenza (H1N1) 2009 hospitalization and effectiveness of pharmaceutical and non-pharmaceutical interventions in its prevention. A case-control study”* [19]. Briefly, this case-control study recruited hospitalized cases older than 6 months with confirmed influenza A (H1N1) 2009 admitted to one of the 36 participant hospitals, of seven Spanish Autonomous Communities, for flu syndrome, respiratory failure, septic shock or multi-organ failure (inpatient). Four control patients, matched by the time period of diagnosis and the region of residency were recruited for each case. One of them was a patient who had consulted a primary care centre for flu syndrome and had confirmed diagnosis of influenza A (H1N1) 2009, but had not required hospitalization (outpatient). Patients with hospital-acquired flu infection and those who did not give informed consent for participation were excluded.

Data analysed in this manuscript was based on a convenience subsample of inpatients and outpatients both with confirmed diagnosis of influenza, from 24 out of the 36 centres which agree to collaborate with the follow-up evaluation; like a nested case-series within a case-control. The decision of including a subsample of patients was based on the sample size estimations (see below) and the resources available for this study. Patients belonging to this subsample and who did not complete follow up were excluded from the analysis. Comparisons between finally included patients and patients of the case control study were done using Chi-square test.

Considering a sample size of 200 patients per group (inpatients and outpatients), with a standard error of €722.3 [1], and an alpha error of 0.05, the precision (width of the 95% confidence interval) of the mean cost would be €200.2.

#### Variables and Sources of information

Information was collected in two moments: at baseline and at follow up. Variables measured at baseline referred to the seven days previous to hospital admission (inpatients) or index medical visit (outpatient). Because this study started after the beginning of the pandemic, this information was gathered retrospectively: median time from the index medical visit or hospital admission to information collection was 125 days (IQR 89–166). Variables then collected included: (a) socio-demographic (sex, age, occupation and Autonomous Community) and clinical characteristics (e.g. presence and type of previous comorbidities), (b) ambulatory healthcare resource utilization such as preventive measures received (e.g. pandemic influenza vaccination in patients recruited after 16th November 2009, when the vaccination campaign started in Spain), medication, laboratory, imaging tests and medical visits due to flu symptoms, (c) inpatients' hospital information regarding unit and length of stay, and (d) work absenteeism of patients and caregivers and need to pay for a caregiver. At this time clinical and hospital variables were gathered from the patient's medical chart, while social and ambulatory healthcare resource utilization was obtained by a face-to-face or telephone interview.

At follow up, information about clinical evolution, additional healthcare resources utilization and work absenteeism, occurring after hospital discharge (inpatients) or index visit (outpatients), was obtained through telephone interview, with a median time from baseline of 105 days (IQR 63–166).

During both, baseline and follow up evaluations, in case of children and adults presenting mental disabilities, a *proxy* was interviewed instead.

While information on the type of medication (e.g. antibiotics, antipyretics) and days of treatment was collected, the specific drug and dose were not available. We assumed utilization rates in accordance with recommendations from local Clinical Practice Guidelines, for adults and children separately [20], [21].

Social class was defined based on occupation and according to the Spanish Society of Epidemiology classification. Categories were then grouped into manual (classes IV y V) and non manual (classes I, II and III) [22].

### Estimating unit costs

The Spanish National Health System does not have information of unit costs. Hence, alternative available sources were considered for the monetary valuation of healthcare utilization. **Table 1** describes the unit costs used in this study as well as the sources of information. Unit costs of ambulatory healthcare resources considered included: (a)the reimbursement tariff available at the public price lists of the official bulletins of Catalonia [23] and Madrid [24] [24]; (b)the retail price of medicines published at a Spanish vademecum [25]; (c)the actual costs of diagnostic tests calculated from the price list of the Hospital Clinic of Barcelona; and (d)the mass media publications of the pandemic vaccine price [26]. The cost per day of hospitalization was calculated in 77 patients (not necessarily included in this study) admitted for influenza A (H1N1) (ICD-9 488.1) to one of the participant hospitals (Hospital del Mar) during the same study period. This hospital has a clinical costing system that allows establishing hospital expenses according to hospitalization unit [27]. The cost per day at the Intensive Care Unit (ICU) and the general ward was calculated separately dividing the total expenses by the total days of hospitalization in each unit.

**Table 1:** Unit costs of healthcare and social^1^ resources: sources of information and calculation method.

*Resource*	*Unit Cost (€)*	*Source*
Primary care GP's visit^2^	37.5	DOGC, BOMA
Primary care ED visit^3^	87.7	DOGC
Home medical care visit^3^	58.5	DOGC
Occupational care visit^3^	100	Expert
Hospital ED visit^2^	139.6	DOGC, BOMA
Outpatient office visit (adults)^2^	167.3	BOMA
Outpatient office visit (children)^2^	217.3	BOMA
Pandemic influenza vaccine^2^	9.7	Mass media
Seasonal vaccine^c^	11.16	Vademecum
Oseltamivir (day of treatment for adults)^4^	6.514	Vademecum
Oseltamivir (day of treatment for children)^4^	3.478	Vademecum
Antibiotics (day of treatment)^4^	1.61	Vademecum
Oral glucocorticoids (day of treatment)^4^	0.62	Vademecum
Ibuprophen-acetaminophen (day of treatment)^4^	0.35	Vademecum
Chest Radiography (front and side views)^5^	21.3	H. Clinic
CT without contrast^5^	120.2	H. Clinic
Laboratory test^5^	9.6	H. Clinic
Cost per day of hospitalization in the ICU^6^	1342.3	PSMar
Cost per day of hospitalization in the in GW of ICU-inpatients^6^	419.7	PSMar
Cost per day of hospitalization in the in GW of general-ward inpatients (children)^6^	630	PSMar
Cost per day of hospitalization in the in GW of general-ward inpatients (adults)^6^	505.2	PSMar
Cost per working day - Andalusia^3^	129.2	INE
Cost per working day - Catalonia^3^	134	INE
Cost per working day - Madrid^3^	137.6	INE
Cost per working day – Community of Valencia^3^	133.1	INE
Cost per working day - Castilla y Leon^3^	129.4	INE
Cost per working day – Basque Country^3^	127.9	INE
Cost per working day - Navarre^3^	127.7	INE

GP: General Practitioner; ED: Emergency Department; CT: Computed Tomography; ICU: Intensive Care Unit; GW: General Ward; DOGC: Diari Oficial Generalitat de Catalunya (Departament de Salut, Resolución SLT/383/2009); BoMA: Boletín Oficial de Madrid (Conserjeria de Salud y Consumo), Order 629/2009); Expert: expert in occupational health; Mass media: pandemic vaccine prices published in “El País” newspaper available at http://www.elpais.com/articulo/sociedad/Francia/vende/excedente/vacunas/gripe/elpepusoc/20100104elpepisoc_4/Tes Vademecum: Vademecum.es. CMP Medicom Editorial, S.A.; H. Clinic: price list of the Hospital Clínic de Barcelona; PSMar: clinical costing system of Parc de Salut Mar. INE: National Institute of Statistics.

1 Social resources analysed in this study included work absenteeism of patients and caregivers and paid-caregiver requirement.

**Methods for unit cost calculation:**

2 Mean cost of available information.

3 Published cost, price or reimbursement tariff.

4 Mean of Recommended Retail Price of the different brands available.

5 Actual cost calculated from price lists applying the correspondent discount.

6 Mean costs per day of hospitalization of inpatients with pandemic influenza A (H1N1) admitted to the Hospital del Mar during the same period.

The cost per working day was obtained from the National Statistics Institute (INE), and was specific for each Autonomous Community on the fourth quarter of 2009 [28].

### Analysis

Descriptive analysis was done to characterize the study population. The number and percentage of patients that used each healthcare and incurred in work absenteeism was informed. Mean utilization frequencies and standard deviation (SD) were calculated among users. Patients' work absenteeism was estimated among those who were working when the disease started.

Direct costs associated to healthcare resource utilization and indirect costs associated to work absenteeism of patients and caregivers (income losses) were estimated by multiplying frequency of use by each unit cost. Mean cost due to each type of resource utilization was calculated among all patients. Pie charts were created to show direct healthcare and indirect costs' distribution in inpatients admitted to de ICU (ICU inpatients), inpatients hospitalized exclusively at the general ward (general-ward inpatients), and outpatients. Comparisons of cost per patient between age groups were performed using Kruskal-Wallis test.

All analyses were performed stratifying by inpatients and outpatients.

#### Sensitivity analysis

Mean and 95%CI for direct healthcare, work absenteeism and total costs per patient were estimated by a multivariate probabilistic sensitivity analysis using Monte Carlo simulation. Analysis was performed separately for outpatients, ICU inpatients, general-ward inpatients and all inpatients. Input variables distribution and parameters were estimated by fitting distribution to data (**table 2**). For the probability distribution of unit costs we considered the available published information mentioned in the unit cost section. Frequency of healthcare utilization and work absenteeism probabilities were based on the observed distribution in our study population. We used a Chi-square test to compare the observed data in our data-set with the potential distributions that could better represent them. The distributions with the lowest Chi- squared value were chosen.

**Table 2:** Statistical characteristics of the input variables included at the multivariate probabilistic sensitivity analysis.

	*Distribution of inputs*				
	Unit Cost	Utilization Frequency			
		Outpatient	Inpatient	General ward patient	ICU patient
**Medical visits**					
Primary care GP's office	Uniform (36–39)	Poisson (1.6652)	Negative binomial (2-0.60777)	Negative binomial (2-0.59519)	Geometric (0.54054)
Home medical care^1^	58.5€	Binomial (1-0.013453)	Geometric (0.80374)	Poisson (0.10294)	Binomial (1-0.15)
Occupational care^1^	100€	Binomial (1-0.079545)	Binomial (1-0.017442)	Binomial (1-0.0073529)	Geometric (0.62329)
Hospital ED	Uniform (118.9–174.6)	Poisson (0.44643)	Geometric (0.72269)	Geometric (0.71958)	Poisson (0.15)
Primary care ED^1^	87.7€	Geometric (0.92946)	Geometric (0.80751)	Geometric (0.77273)	
Outpatient office	Uniform (94–251)	Binomial (1-0.022523)	Geometric (0.68526)	Geometric (0.69036)	Geometric (0.8)
**Medication**					
Antiviral agents	6.5 (adults), 3.5 (children)	Binomial (1-0.30952)	Geometric (0.27636)	Geometric (0.25207)	Geometric (0.47222)
Antipyretics^1^	0.35€	Geometric (0.39051)	Geometric (0.21287)	Geometric (0.21053)	Geometric (0.22472)
Antibiotics^1^	1.61€	Negative binomial (1-0.35484)	Negative binomial (1-0.23465)	Geometric (0.23735)	Geometric (0.21739)
Glucocorticoids^1^	0.62€	Geometric (0.94872)	Geometric (0.38826)	Geometric (0.40356)	Geometric (0.26667)
**Vaccines**					
Pandemic influenza (H1N1) 2009	Uniform (9.34–10)	Binomial (1-0.026786)	Binomial (1-0.11047)	Binomial (1-0.10294)	Binomial (1-0.15)
Seasonal influenza^1^	11.2€	Binomial (1-0.27232)	Binomial (1-0.36628)	Binomial (1-0.36029)	Binomial (1-0.35)
**Diagnosis Tests**					
Radiography^1^	21.3€	Binomial (4-0.064103)	Geometric (0.67143)	Poisson (0.44737)	Binomial (1-0.4)
Computed Tomography^1^	120.2€	Binomial (1-0.010101)	Geometric (0.90476)	Poisson (0.064935)	
Laboratory^1^	9.6€	Binomial (2-0.13452)	Geometric (0.73438)	Geometric (0.73786)	Binomial (1-0.2)
**Hospitalization** (day)					
Intensive Care Unit	Uniform (1209.9–1497.81)		Geometric (0.4984)		Poisson (7.85)
General Ward	Beta General (0.89803-2.1841-330.79-1102.29)		Negative binomial (3-0.34978)	Poisson (4.9926)	Poisson (9.6)
**Work Absenteeism** (day)					
Patients	Uniform (126.05–139.25)	Geometric (0.15581)	Geometric (0.10935)	Geometric (0.11175)	Geometric (0.11801)
Caregivers	Uniform (126.05–139.25)	Geometric (0.74172)	Geometric (0.29706)	Geometric (0.41159)	Geometric (0.11801)
**Paid caregiver** (day)	Uniform (126.05–139.25)	Poisson (0.066964)	Poisson (0.098214)	Poisson (0.066964)	

GP: general practitioner; ED: emergency department.

Geometric distribution parameter: p (probability of success in each trial).

Uniform distribution parameters: minimum, maximum.

Negative Binomial parameters: s - p (s = mean number of successes, p = probability of success in each trial).

Binomial parameters: n - p (n = number of trials, p = probability of success in each trial).

Beta General Distribution parameters: alpha1- alpha2 – minimum - maximum (alpha1 and alpha 2 = shape parameters).

Poisson parameter: λ (λ = mean number of success).

1 no variability was introduced to these inputs due to lack of alternative unit cost information availability.

The main factors affecting cost per patient were identified in the estimated model by calculating Spearman's rank correlation coefficients as a measure of the magnitude of the association between each variable and the outcome.

The @Risk 5.0. add-in tool to Microsoft Excel was used for the sensitivity analysis, (Palisade Corporation, Ithaca, NY, SA); and SPSS 13.0 for the rest of the analyses(SPSS, Inc., Chicago, IL, USA).

#### Estimating the healthcare economic burden of patients with influenza A (H1N1) 2009 in Spain

To estimate the healthcare economic burden generated by patients with pandemic influenza A (H1N1) 2009, we considered the mean healthcare-cost per patient obtained in the sensitivity analysis for each type of patient and the number cases reported in Spain. The results of ICU inpatients and general-ward inpatients were estimated separately and then summed up to obtained the inpatients' overall impact. At a national level, 852 and 2,173 inpatients with confirmed diagnosis of influenza were attended at ICU and general-ward, respectively [10]. Government institutions also estimated a total of 744,795 confirmed outpatients among the 1,414,000 estimated clinical cases [9]. We informed the economic impact on healthcare system for both, estimated confirmed and clinical cases.

## Results

**Figure 1:**
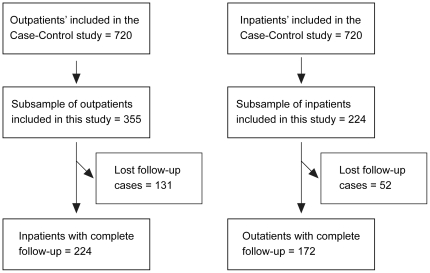
Sample selection flow chart.

One hundred seventy two inpatients and 224 outpatients were included in the study. Neither groups differed statistically from those of the case control study, regarding the distribution of sex (p = 0.91), age group (p = 0.13) and employment before flu (p = 0.33) (**figure 1**).

Temporal distribution of cases included in our study was comparable to that reported in Spain. First circulation of the virus was detected in Spain on week 21/2009 (April 24th to May 30th) and the beginning of pandemic wave on week 40 (October 4th to 10th). The first patient included in the study was recruited on August 1st. Peak activity was observed on week 46/2009 in both, Spanish surveillance system and our study [9].

Patient's characteristics are presented in **table 3**. Mean age of both groups was below 25, and less than 10% were over 65 years old. Sixty four percent of inpatients and the 50.3% of outpatients had some previous comorbidity. Most patients were in working age (61% and 73.7% for inpatients, and outpatients, respectively). Of those, 43.8% of inpatients and 66.1% of outpatients were currently working. Pandemic vaccine was applied to 24.7% inpatients and 8% of outpatients recruited after 16^th^ November 2009.

**Table 3:** Patients' characteristics.

	*Inpatients*	*Outpatients*
	n = 172	n = 224
	n (%)	n (%)
**Sex**		
Female	88 (51.2%)	131 (58.5%)
Male	84 (48.8%)	93 (41.5%)
**Age**, mean (SD)	23.3 (1.8)	19.5 (1.3)
≤4	21 (12.2%)	20 (8.9%)
5–16	30 (17.4%)	28 (12.5%)
17–64	105 (61%)	165 (73.7%)
≥65	16 (9.3%)	11 (4.9%)
**Autonomous Community**		
Andalusia	39 (22.7%)	14 (6.3%)
Castile and León	1 (0.6%)	0 (0%)
Catalonia	56 (32.6%)	60 (26.8%)
Basque Country	47 (27.3%)	72 (32.1%)
Madrid	14 (8.1%)	70 (31.3%)
Valencian Community	6 (3.5%)	0 (0%)
Foral Community of Navarre	9 (5.2%)	8 (3.6%)
**Employment before flu in adults in working age**	46/105 (43.8%)	109/165 (66.1%)
*Missing values*		*1 (0.6*%*)*
**Social Class**		
Non-manual workers	72 (41.9%)	145 (64.7%)
Manual workers	51 (29.7%)	48 (21.4%)
*Missing values*	*49 (28.5%)*	*31 (13.8%)*
**Comorbidities**		
Any comorbidity	110 (64%)	113 (50.3%)
Respiratory	57 (33.1%)	54 (24.1%)
Neuromuscular	21 (12.2%)	7 (3.1%)
Cardiovascular	47 (27.3%)	27 (12.1%)
Diabetes or obesity	36 (20.9%)	31 (13.8%)
Immunosuppressive condition	28 (16.3%)	20 (8.9%)
Chronic Renal Failure	11 (6.4%)	8 (3.6%)
Rheumatologic	1 (0.6%)	7 (3.1%)
**Pregnancy in women in childbearing age**	9/46 (19.6%)	31/88 (35.2%)
*Missing values*	*2 (4.4*%*)*	*7 (8*%*)*
**Pandemic influenza H1N1 vaccination** 1	19 (24.7%)	6 (8%)
**Seasonal influenza vaccination**	63 (36.6%)	61 (27.2%)
**Social isolation measures**	6 (3.4%)	4 (1.7%)
**Preventive mask use**	53 (30.8%)	89 (39.7%)

1 Among 77 inpatients and 75 outpatients who we recruited after the pandemic (H1N1) 2009 vaccine was available in Spain (16^th^ November 2009).

Utilization of healthcare and social resources is shown in **table 4**. The mean (SD) frequency of use of each resource is described exclusively among users. Before admission, inpatients tended to seek medical assistance at the primary care general practitioner's office (43%) or at the hospital emergency department (22.7%); while, after discharge, they were also frequently assisted at the hospital outpatient office (23.8%). Almost twenty percent (18.6%) of inpatients received ambulatory treatment with antiviral drugs before hospitalization. In addition, 39.5% were treated with antibiotics at that time. Twenty inpatients (12.8%) required hospitalization at the ICU. The total hospital length of stay was 17.4 days (SD = 9.2) for these patients, while general-ward inpatients were hospitalized for 5 days on average (SD = 4.4).

**Table 4:** Utilization estimators of healthcare and social^1^ resources among users.

	*Inpatients*						*Outpatients*	
	n = 172						n = 224	
	Before admission		After discharge		Total			
	Patients	Use frequency	Patients	Use frequency	Patients	Use frequency	Patients	Use frequency
	n (%)	Mean (SD)	n (%)	Mean (SD)	n (%)	Mean (SD)	n (%)	Mean (SD)
**Medical Visits** (number of visits)								
Primary care GP's office	74 (43%)	1.5 (0.9)	63 (36.6%)	1.7 (1)	104 (60.5%)	2.1 (1.3)	224 (100%)	1.7 (1.3)
Home medical care	15 (8.7%)	1.1 (0.4)	3 (1.7%)	8.3 (7)	17 (9.9%)	2.5 (4)	3 (1.3%)	1 (0)
Occupational care	2 (1.2%)	1 (0)	1 (0.6%)	1 (0)	3 (1.7%)	1 (0)	14 (6.3%)	1 (0)
Hospital ED (without admission)	39 (22.7%)	1.3 (0.5)	7 (4.1%)	2 (1)	45 (26.2%)	1.5 (0.9)	85 (37.9%)	1.2 (1)
Primary care ED	24 (14%)	1.5 (0.7)	3 (1.7%)	2 (1.7)	26 (15.1%)	1.6 (1.1)	12 (5.4%)	1.4 (1.2)
Outpatient office	5 (2.9%)	1.2 (0.4)	41 (23.8%)	1.8 (1.2)	44 (25.6%)	1.8 (1.2)	6 (2.7%)	1 (0)
**Ambulatory medication** (days of treatment)								
Antiviral agents	32 (18.6%)	4.6 (1.8)	52 (30.2%)	5.2 (3.4)	70 (40.7%)	5.7 (4)	58 (25.9%)	5.7 (1.4)
Antipyretics	130 (75.6%)	2.8 (2.8)	42 (24.4%)	7 (4.6)	134 (77.9%)	4.7 (4.4)	192 (87.5%)	5.8 (4)
Antibiotics	68 (39.5%)	3 (3.1)	51 (29.7%)	7.4 (4.6)	87 (50.6%)	6.4 (4.9)	46 (20.5%)	8.7(2.8)
Glucocorticoid	37 (21.5%)	3.1 (4.4)	14 (8.1%)	11 (14.3)	45 (26.2%)	6 (10)	2 (0.9%)	6 (0)
**Vaccines** (doses)								
Pandemic influenza A (H1N1)^2^	19 (24.7%)	1 (0)			19 (24.7%)	1 (0)	6 (8%)	1 (0)
Seasonal influenza	63 (36.6%)	1 (0)			63 (36.6%)	1 (0)	61 (27.1%)	1 (0)
**Ambulatory diagnosis tests** (number of tests)								
Radiography			32 (18.6%)	1.4 (1)	32 (18.6%)	1.4 (1)	47 (21%)	1.1 (0.3)
Tomography			6 (3.5%)	1.7 (0.8)	6 (3.5%)	1.7 (0.8)	2 (0.9%)	1 (0)
Laboratory			21 (54.7%)	1.6 (0.9)	21 (54.7%)	1.6 (0.9)	52 (23.2%)	1 (0.2)
**Hospitalization** (days)								
Hospitalization at ICU					20 (12.8%)	7.8 (3.7)		
Hospitalization at GW of ICU-inpatients					20 (12.8%)	9.6 (7.7)		
Hospitalization at GW of general-ward inpatients					136 (87.2%)	5 (4.4)		
**Work absenteeism** (days)								
Absenteeism in working patients					46/46 (100%)	30.5 (20.7)	100/109 (91.7%)	9 (6.3)
Patients whose caregivers required work absenteeism					38/172 (21.7%)	10.7 (14.1)	19/224 (8.5%)	4.1 (4.1)
**Patients who paid for a caregiver** (days)					3/172 (3%)	5.5 (5.8)	1/224 (0.4%)	15 (0)

GP: general practitioner, ED: emergency department; ICU: Intensive Care Unit; GW: General Ward.

*Missing value range:* Medical visits = 0 to 17, Medication = 0 to 19, *Missing value*: hospitalization = 16.

1 Social resources analysed in this study included work absenteeism of patients and caregivers and paid caregiver.

2 Among 77 inpatients and 75 outpatients who we recruited after the pandemic (H1N1) 2009 vaccine was available in Spain (16^th^ November 2009).

More than 90% of the patients in the labour force had to be absent from work due to the flu. Mean number of lost working days was 30.5 for inpatients and 9 for outpatients. Almost nine percent (8.5%) of outpatients needed a caregiver who had to interrupt work. This percentage ascended to 21.7% in the case of inpatients.

**Table 5** shows direct healthcare, indirect and total costs per patient. For inpatients, direct costs associated to ambulatory healthcare utilization and indirect costs derived from work absenteeism were estimated among 172 patients. However, hospital and total costs could only be calculated among 156 patients that had complete information of length of hospital stay. The total mean cost was €6,028 per inpatient and €749.2 per outpatient. Medical visits accounted for the majority of ambulatory costs in both groups of patients. The mean cost for hospitalization was €4,438.5. Inpatients' mean direct healthcare cost (€4,687.5) exceeded mean indirect cost (€1,383). The opposite was observed in the case of outpatients (indirect costs = €582, direct healthcare costs = €167.3).

**Table 5:** Direct healthcare and indirect costs per patient.

	*Cost per Inpatient*	*Cost per Outpatient*
	n = 172	n = 224
	Mean (SD)	Mean (SD)
**DIRECT HEALTHCARE COST**		
**Ambulatory resources**		
Medical visits	218.9 (241.5)	142.1 (133.8)
Medication	21 (25.8)	13.8 (17.9)
Vaccines	5.2 (7.1)	3.3 (5.3)
Diagnosis tests	14.6 (54.1)	8.1 (17.9)
**Hospitalization** 1		
ICU	1181.3 (3451.6)	
General ward	3257.2 (3057.7)	
**INDIRECT COST** 2		
Patient absenteeism	1068.6 (2267.1)	534 (814.3)
Caregiver absenteeism	311.7 (1044.8)	45.7 (211)
**TOTAL COST**		
**Direct healthcare cost**		
Ambulatory cost	259.7 (273.9)	167.3 (148)
Hospital cost^1^	4438.5 (5714.8)	
Total direct cost^1^	4687.5 (5729.3)	167.3 (148)
**Indirect cost** 2	1383 (2421.2)	582 (852.8)
**Total cost** 1	6028 (6250.8)	749.2 (885.8)

ICU: Intensive Care Unit.

1 Inpatients hospital and total costs were estimated over 156 inpatients with complete information about length of stay.

2 Indirect cost is derived from work absenteeism of patient and caregiver and paid-caregiver requirement.

Mean cost per patient differed between age groups. The highest inpatient's cost was observed in individuals over 65 years old (€7,985 —SD = 10,979), comparing with the younger groups: ≤4 years: €5026 (SD = 3,996), 5 to 16 years: €3,736 (SD = 4,685), 17 to 64 years: €6664 (SD = 6,059) (p = 0.014). By contrast, cost per outpatient was highest among patients in working age (€944 —SD = 947). Mean cost per outpatient of other age groups were estimated as follows: ≤4 years: € 308 (SD = 416), 5 to 16 years: €150 (SD = 128), ≥65 years: €273 (SD = 178) (p<0.001).

**Figure 2:**
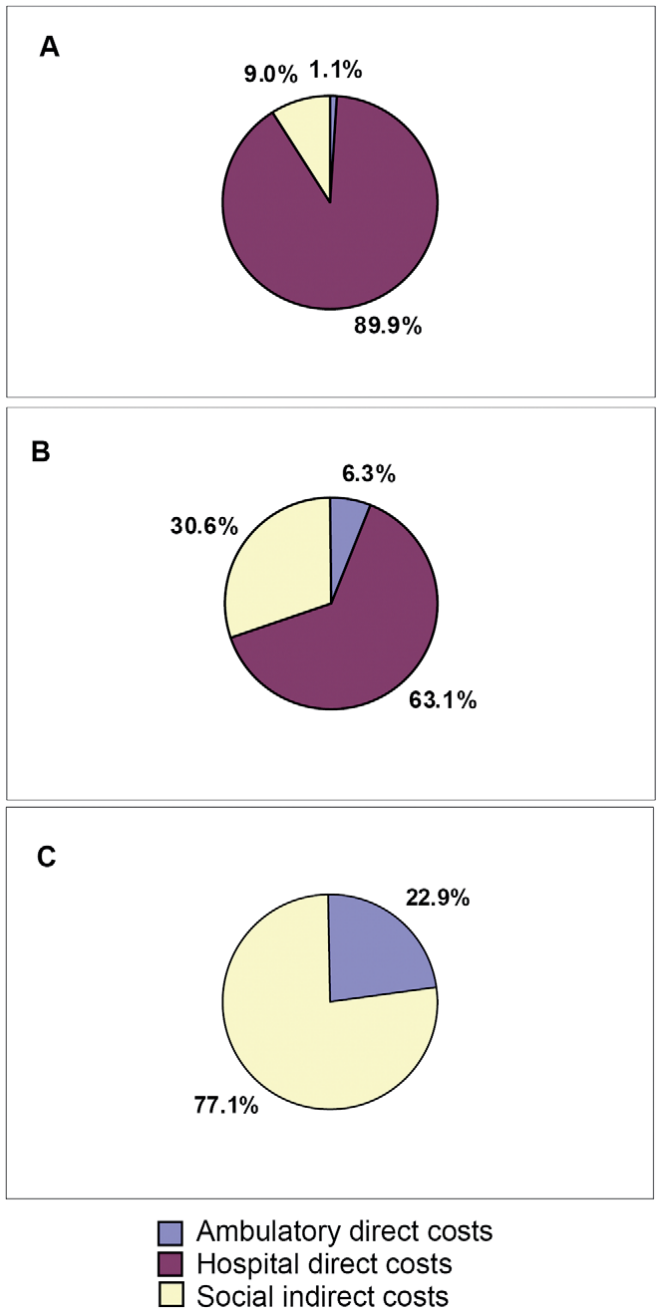
Direct healthcare and indirect^1^ costs' distribution according to patient's group. ^1^Indirect cost is derived from work absenteeism of patient and caregiver and paid caregiver. A. Inpatients admitted to the intensive care unit at any time of the hospitalization. (n = 20). B. Inpatients hospitalized exclusively at the general ward. (n = 136). C. Outpatients. (n = 224).

Almost 90% of total cost per ICU-inpatient was explained by hospital related costs. Indirect costs for these patients accounted for 9% of total costs. By contrast, the proportion of indirect cost for general-ward inpatients was 30%. This percentage ascended to 77% in the case of outpatients (**figure 2**).

### Sensitivity Analysis

**Figure 3:**
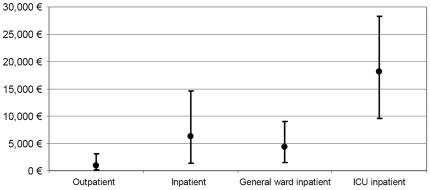
Mean cost per patient and its 95%CI based on the sensitivity analysis.

Estimates of mean cost and their intervals obtained by the sensitivity analysis varied significantly across patients groups (**figure 3**). Mean cost per inpatients was €6,236 (95%CI 1,384–14,623). Among these, cost was greatest for those needing care at the ICU (Mean = €18,095, 95%CI 9,634–28,333), while mean cost per general-ward inpatient was €4,288 (95%CI 1,430–8,957). For outpatients, mean cost was €940 (95%CI 66–3,064).

**Figure 4:**
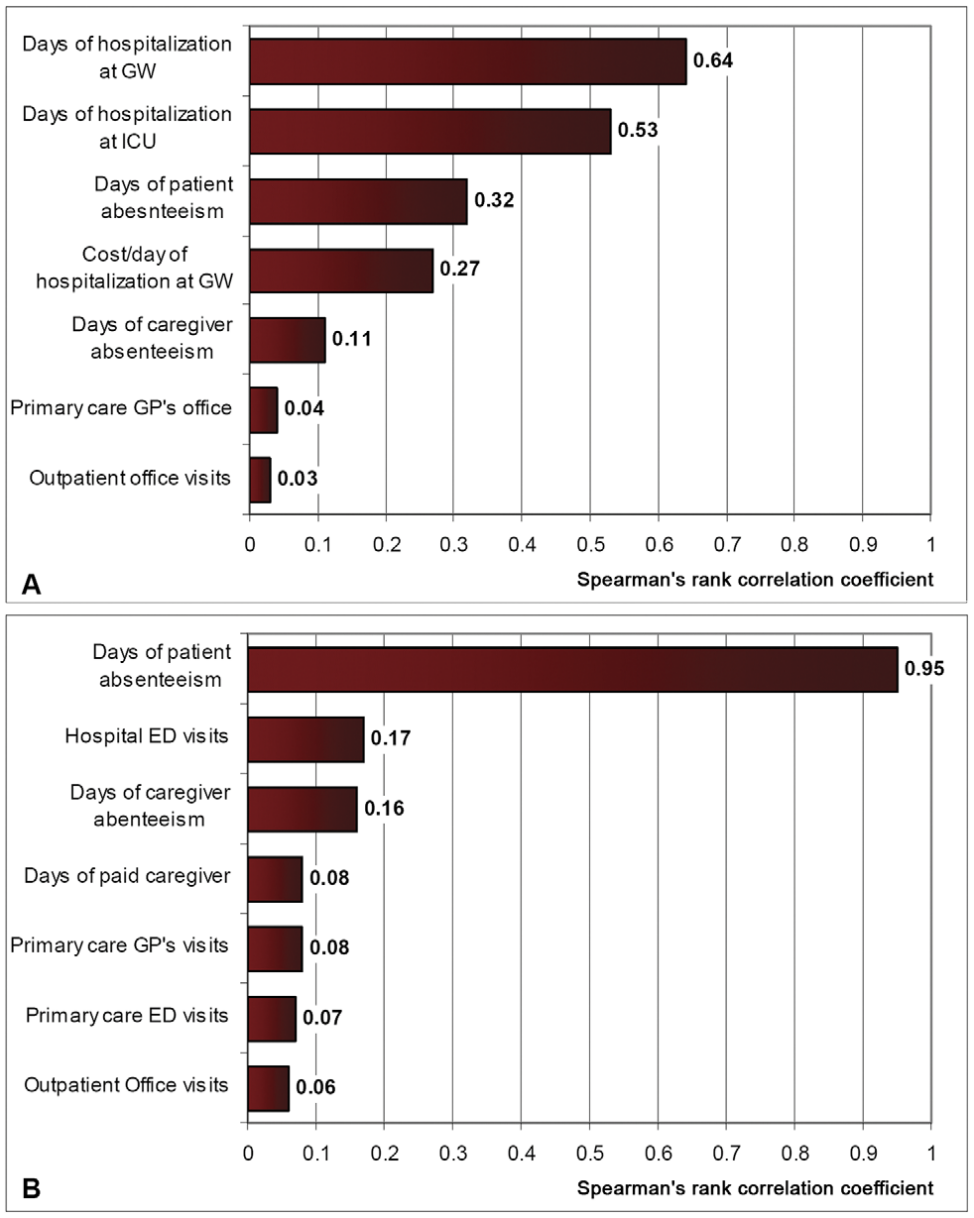
Correlation of cost per patient and the main input variables (Spearman's rank). A. Inpatients. B. Outpatient. Abbreviations: GW: General Ward, ICU: Intensive Care Unit, GP: General Practitioner, ED: Emergency Department.

The sensitivity analysis also identified the variables in the model to which the estimates were most sensitive (**figure 4**). For inpatients, the most correlated variables with total mean cost was (1) the length of stay at the general ward (*ρ* = 0.64), (2) the length of stay at the ICU (*ρ* = 0.53), and (3) the length of patient's work absenteeism (*ρ* = 0.32). On the other hand, outpatients' mean cost was mostly correlated to length of patient's absenteeism (*ρ* = 0.95).

### Healthcare economic burden of patients with influenza A (H1N1) 2009 in Spain

Considering the number of confirmed pandemic influenza A (H1N1) 2009 cases in Spain and the results of healthcare cost per patient obtained in the sensitivity analysis, the economic impact on the health services in Spain was €144,773,577 (95%CI 13,753,043–383,467,535). Up to 86% of this cost was be generated by outpatients, while the remaining 14% would be attributable to inpatients' utilization. Assuming that mean costs associated to clinical (non-confirmed) flu cases were similar to those of patients with confirmed diagnosis of influenza A (H1N1), the social economic burden was estimated in €256,530,812, (95%CI 18,437,478–694,647,860).

## Discussion

Based on primary data of patients with confirmed diagnosis of 2009 pandemic influenza A (H1N1) 2009, we have estimated the pattern of healthcare resources utilization as well as patient and caregiver work absenteeism. Derivate cost per patient and the economic impact on healthcare services were described. Mean length of hospital stay was almost fourfold for patients requiring critical care (ICU) than for patients treated at the general ward only. As expected, inpatients needed more time to recover and their mean length of work sick leave was 30.5 days, in comparison with the 9 days observed for outpatients. Loss of productivity was also important for caregivers, since 21.7% and 8.5% of inpatients and outpatients respectively had caregivers who had to be absent from work. From an economic point of view, at an individual level, inpatients had a greater cost (€ 6,236 per inpatient) than outpatients (€ 940 per outpatient). From the healthcare provider's perspective, the 86% of economic national burden was the result of outpatients' resource utilization. The healthcare economic burden of patients with confirmed diagnosis of influenza A (H1N1) 2009 was estimated in €144,773,577, and €256,530,812 when considering clinical cases.

The description of healthcare utilization is essential for the healthcare provider's administration, the economic evaluation of health technologies and the estimation of the burden of a disease. In agreement with previous studies, length of hospital stay of general-ward inpatients doubled that observed for seasonal flu [6], [10]. It has been described that the risk of serious complications was not elevated in patients with pandemic influenza (H1N1) 2009 compared with recent seasonal strains [29]. Thus, longer hospitalizations might be attributed to differences in medical practice during a pandemic outbreak when clinical evolution was uncertain.

Regarding ambulatory healthcare, several economic evaluation studies have included in their models evidence of previous seasonal surges or from patients with influenza-like illness [30], [31]. Our study adds a detailed description of ambulatory healthcare resource utilization, including less frequently used medical assistance (such as home medical visits or occupational care visits) also relevant for the health services organization.

As in previous studies of seasonal influenza, antibiotics were more frequently used than antiviral drugs outside the hospital [32]. Since influenza is a viral infection, the antibiotic prescription was probably inappropriate in most cases. Nevertheless, neither has the existing evidence demonstrated a clear benefit of antiviral drugs in reducing influenza complications [33], [34].

Productivity losses represented the most important impact of the pandemic outbreak. Compared with estimations for seasonal influenza, length of work sick leave was almost ten times higher in the case of inpatients, and at least two times higher in the case of outpatients [11], [35]. Moreover, length of work absenteeism during the pandemic in Spain exceeded that reported in other European countries [36]. This was not an exclusive feature of the pandemic influenza A (H1N1), since it was also observed in other pathologies [37].

Costs per patient derived from our study did not differ much from previous estimates for seasonal influenza in our country. If we consider that 99.8% of clinical cases were outpatients, 0.15% were general-ward inpatients and 0.06% were ICU-inpatients, the mean cost per pandemic influenza A (H1N1) patient in Spain would be €954 [9], [10]. In the same country, Badia et al. calculated the mean cost per patient with seasonal influenza-like illness to be €542.1 (95%CI = 487.1–597.1). This cost and their reported cost due to work leave resembled our results, considering that their study was conducted one decade ago [1]. Also, our estimated direct costs per pandemic inpatient were also similar to those reported for seasonal influenza in the US, which ranged from US$ 2,785 and US$ 13,159 [2], [6], [7], [38].

The economic burden of health services estimated here was considerably low in comparison with previous reports for seasonal influenza in the United States [13], [38]. Molinari et al estimated the direct costs of the annual medical treatment for influenza in U$S 10.4 billion. Although they considered also non-medically attended cases, the cost of these patients accounted for less than 1% of the medical expenditures. In contrast to our results, they calculated that 52% of the expenditures on influenza were attributable to hospitalizations [13]. These differences might be due to the mildness of the influenza H1N1 pandemic, the higher cost per patient considered in their analysis and the methodology used. Another study that included only hospital and emergency costs, estimated the annual cost burden at $44 to $163 million [38]. Also, costs of pandemic influenza A (H1N1) 2009 were clearly lower than direct medical costs associated to other pathologies in Spain, such as metabolic syndrome (€1,900 million) and knee and hip osteoarthritis costs (€4,075 million) [39], [40].

### Limitations

The results of this study should be interpreted taking into account several limitations. First, the study population was a subsample of patients recruited for a case-control study. This is important for outpatients who were selected to be matched with inpatient rather than a representative sample of the outpatient Spanish population. Therefore, external validity could be compromised. However, patient's were temporally representative of the pandemic surge in Spain [9], and the prevalence of comorbidities among our inpatient's was similar to that reported before [10], [41]. Also, although follow-up response was not 100%, demographic characteristics of lost patients and those who stayed in the study were not statistically different. Second, none of the patients included died during the influenza infection. Consequently, our estimates underestimate the actual impact of the pandemic. Nevertheless, most patients who died during the pandemic were either old or had previous severe chronic conditions, thus not affecting much our estimation of indirect costs [10], [41]. Third, we could only analyse flu cases that had contact with health services and were laboratory confirmed. This might have prevented us from overestimating costs due to over diagnosis of influenza. However, it could have lead us underestimate productivity costs among specific populations (housekeepers, or non-contracted individuals, for instance) [42]. Fourth, even though there is evidence regarding possible differences in mean cost by social class [12], comparisons stratifying by this variable could not be performed due to many missing values. Also, we need to indicate that the number of cases that were used to estimate the costs per ICU-inpatient was small (n = 20). Finally, the limitations related with the sources of data used in our study deserve a comment. Although some of the information was directly gathered from the patient during an interview (to patients or proxies) and in some cases several months after the flu, memory bias was probably minor due to the influenza pandemic's important mass media repercussion [43]. We had to consider alternative sources of information for unit costs, as there is no accepted common information source for the Spanish national healthcare system. While the source of unit costs for hospital and day absenteeism were reliable, many ambulatory unit costs were probable overestimated because they were obtained from the list of prices of health services provision to third parties. It is worth mentioning that several of the limitations listed above were addressed by the sensitivity analyses performed, because variations in unit costs, hospitalization length of stay in each area and days of work absenteeism were introduced as inputs of the model. The resulting confidence intervals of estimates represent the degree of uncertainty introduced by these limitations.

In conclusion, this paper provides information for health service providers and society during the pandemic influenza A (H1N1) 2009 which might be of interest for sizing the costs and needs of care in such episode, and in future outbreaks of similar epidemiological characteristics. Work absenteeism of patients and caregivers accounted for the majority of costs per outpatient. Interventions such as home-care provided by health services should be explored to reduce parents' and other relatives' productivity losses. Clinical Practice Guidelines for general practitioners might reduce economic impact on health services by minimizing unnecessary health resource utilization in a pandemic scenario. In addition, the evidence given by this study, together with other global expenses, such as prevention campaigns, massive vaccine purchase and other economy costs of reduced productivity, should be useful to evaluate the global impact of the pandemics.
